# Multiplex Bead Array Assay of a Panel of Circulating Cytokines and Growth Factors in Patients with Albuminuric and Non-Albuminuric Diabetic Kidney Disease

**DOI:** 10.3390/jcm9093006

**Published:** 2020-09-18

**Authors:** Vadim V. Klimontov, Anton I. Korbut, Nikolai B. Orlov, Maksim V. Dashkin, Vladimir I. Konenkov

**Affiliations:** Research Institute of Clinical and Experimental Lymphology—Branch of the Institute of Cytology and Genetics, Siberian Branch of Russian Academy of Sciences, 630060 Novosibirsk, Russia; anton.korbut@gmail.com (A.I.K.); nbo700@mail.ru (N.B.O.); mdashkin@invitro.ru (M.V.D.); vikonenkov@gmail.com (V.I.K.)

**Keywords:** type 2 diabetes, chronic kidney disease, glomerular filtration rate, albuminuria, inflammation, fibrosis, cytokines, growth factors

## Abstract

A panel of cytokines and growth factors, mediating low-grade inflammation and fibrosis, was assessed in patients with type 2 diabetes (T2D) and different patterns of chronic kidney disease (CKD). Patients with long-term T2D (N = 130) were classified into four groups: no signs of CKD; estimated glomerular filtration rate (eGFR) <60 mL/min/1.73 m^2^ without albuminuria; albuminuria and eGFR ≥60 mL/min/1.73 m^2^; albuminuria and eGFR <60 mL/min/1.73 m^2^. Thirty healthy subjects were acted as control. Twenty-seven cytokines and growth factors were assessed in serum by multiplex bead array assay. Serum hs-CRP, urinary nephrin, podocine, and WFDC2 were measured by ELISA. Patients with T2D showed elevated IL-1Ra, IL-6, IL-17A, G-CSF, IP-10, MIP-1α, and bFGF levels; concentrations of IL-4, IL-12, IL-15, INF-γ, and VEGF were decreased. IL-6, IL-17A, G-CSF, MIP-1α, and bFGF correlated negatively with eGFR; IL-10 and VEGF demonstrated negative associations with WFDC2; no relationships with podocyte markers were found. Adjusted IL-17A and MIP-1α were predictors of non-albuminuric CKD, IL-13 predicted albuminuria with preserved renal function, meanwhile, IL-6 and hsCRP were predictors of albuminuria with eGFR decline. Therefore, albuminuric and non-albuminuric CKD in T2D patients are associated with different pro-inflammatory shifts in the panel of circulating cytokines.

## 1. Introduction

Diabetes is the most common cause of end-stage renal disease (ESRD) worldwide [1,2]. Recent studies indicate a change in the natural history of chronic kidney disease (CKD) in diabetes. Specifically, normoalbuminuric chronic kidney disease (NA-CKD), which is characterized by a decline in the estimated glomerular filtration rate (eGFR)in the absence of a preceding or accompanying elevation of albuminuria is becoming predominant variant of renal impairment in patients with type 2 diabetes(T2D) [3,4]. A growing body of evidence indicates that albuminuric and non-albuminuric CKD patterns are different in their clinical course and pathophysiology [3,4,5,6].

Chronic low-grade inflammation and activation of fibrogenic pathways are considered as essential elements in development and progression of diabetic kidney disease. Diabetic nephropathy is associated with both systemic and renal inflammation that involves inflammatory cells, cytokines, and growth factors [7,8,9]. In experimental and clinical diabetes, accumulation of inflammatory cells—mostly macrophages—was observed in the renal interstitium [10,11,12], andenhanced expression of pro-inflammatory cytokineswas revealed in the kidneys [13,14,15]. In patients with diabetic nephropathy, interstitial inflammation and fibrosis were found to be histological predictors of ESRD [16]. It was demonstrated that the presence of CKD changes the spectrum of circulating cytokines in patients with T2D [17,18]. Some of specific circulating inflammatory proteins, such as tumor necrosis factor receptor superfamily members, were described as associated with ESRD in patients with type 1 and type 2 diabetes in the recent prospective study [19]. However, the features of cytokine profile in diabetic patients with different CKD patterns have not been studied yet.

The difficulty in assessment of the low-grade inflammation regulation in clinical settings is determined by the variety of secreted molecules and their interfering effects. It is well known that cytokines act in multiple ways with feedback loops, composing the cytokine networks [20]. Therefore, the measurement of individual regulators may not provide a comprehensive view and assessment of a panel of inflammatory regulatorsis preferable. The multiplex platforms, widely used to measure multiple biomarkers from a single analysis, may be a reasonable alternative to single-reaction-based detection methods in such situations [21,22].

Accordingly, the aim of our study was to assess a panel of circulating cytokines and growth factors, mediating low-grade inflammation and fibrosis, by multiplex bead array assay in patients with T2D and different patterns of CKD.

## 2. Materials and Methods

### 2.1. Design

**Figure 1:**
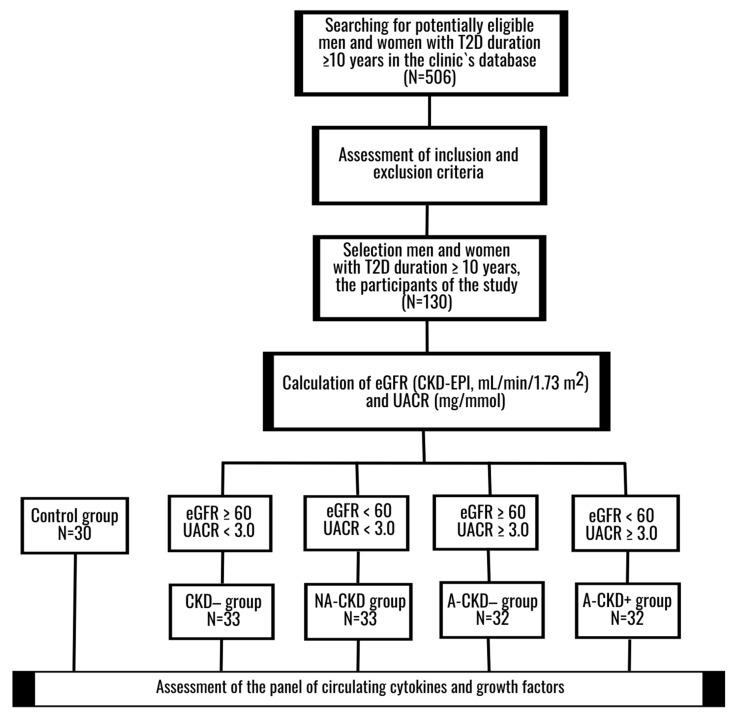
The study design. The panel of circulating cytokines and growth factors was assessed in fourgroups of T2D patients and healthy controls. CKD−: group of patients with estimated glomerular filtration rate (eGFR) ≥60 mL/min/1.73 m^2^ and urinary albumin-to-creatinine ratio (UACR) <3.0 mg/mmol; NA-CKD: non-albuminuric chronic kidney disease, group of patients with eGFR <60 mL/min × 1.73 m^2^ and UACR <3.0 mg/mmol; A-CKD−: group of patients with albuminuria (UACR ≥3.0 mg/mmol) and eGFR ≥60 mL/min/1.73 m^2^; A-CKD+: albuminuric chronic kidney disease, group of patients with eGFR <60 mL/min/1.73 m^2^ and UACR ≥3.0 mg/mmol; eGFR, estimated glomerular filtration rate; T2D, type 2 diabetes; UACR, urinary albumin-to-creatinine ratio.

Caucasian adult male and female patients with known T2D duration for at least 10 years were included in this cross-sectional comparative descriptive study (Figure 1). Non-diabetic CKD, ESRD, urinary tract infection, current diabetic ketoacidosis or hyperglycemic hyperosmolar state, malignant neoplasm, chronic inflammatory or autoimmune diseases in medical history, treatment with anti-inflammatory drugs or antihyperglycemic agents with known antialbuminuric effect (dipeptidyl peptidase-4 inhibitors, glucagon-like peptide-1 (GLP-1)receptor agonists and/or sodium-glucose cotransporter-2 (SGLT-2) inhibitors) for three months prior to inclusion, were considered as the principal exclusion criteria.

Patients were selected from institutional database and classified into four groups, depending on CKD status. Individuals with estimated glomerular filtration rate (eGFR) ≥60 mL/min/1.73 m^2^ and urinary albumin-to-creatinine ratio (UACR) <3.0 mg/mmol were recorded as CKD− group. Those with eGFR <60 mL/min × 1.73 m^2^ and UACR <3.0 mg/mmol were assigned to NA-CKD group. Patients with eGFR ≥60 mL/min/1.73 m^2^ and UACR ≥ 3.0 mg/mmol were defined as albuminuric with preserved renal function (A-CKD−) group. Individuals with eGFR <60 mL/min/1.73 m^2^ and UACR ≥3.0 mg/mmol comprised the albuminuric CKD group (A-CKD+ group). Non-diabetic subjects, matched by sex and age, acted as control; the same exclusion criteria as those for diabetic patients were applied for these individuals.

All patients underwent detailed clinical examination with assessment of diabetes control and in-depth screening/monitoring of complications. The panel of circulating cytokines and growth factors was assessed by multiplex bead array assay. Urinary concentrations of nephrin and podocin, two biomarkers of podocyte injury [23,24,25] and whey acidic protein four-disulfide core domain protein 2 (WFDC2), a biomarker of tubulointerstitial fibrosis [26,27], were determined by the enzyme-linked immunosorbent assay(ELISA).

### 2.2. Ethical Principals

The study protocol was approved by institutional Ethical Committee (Protocol 88, November 22, 2012). All subjects provided their written informed consent prior to inclusion.

### 2.3. Participants

One-hundred and thirty T2D patients, 48 men and 82 women, 42–88 years of age (median 66 years), were included in the study. The duration of T2D varied from 10 to 45 years (median 14 years). The median hemoglobin A_1C_ (HbA_1C_) level was 8.7%, or 72 mmol/mol. The detailed clinical characteristic of patient groups is presented in the Results section. Thirty individuals who had no history of diabetes, obesity, or cardiovascular disease, 11 men and 19 women, 42–84 years of age (median 64 years), acted as controls.

For logistic regression analysis, patients with T2D were divided into discovery and validation cohorts randomly in a ratio of 2:1 (N = 86 and N = 44, respectively). The clinical characteristics of these cohorts are presented in Table A1 in Appendix A.

### 2.4. Methods

The levels of HbA_1C_, total, low-density lipoprotein (LDL)and high-density lipoprotein (HDL) cholesterol, triglycerides, uric acid, and creatinine were assessed with AU480 Chemical Analyzer (Beckman Coulter, Brea, CA, USA) and commercially available cartridges. The eGFR was calculated by the CKD-EPI creatinine formula (2009). Urinary albumin was determined in three morning urine samples by immunoturbidimetry with MindRay Chemical Analyzer (MAL0304, Mindray Medical International Limited, Shenzhen, China); the results were adjusted to excreted creatinine. Concentrations of fibrinogen, soluble fibrin monomer complex (SFMC) and D-dimer were evaluated with hemostasis analyzer system (Instrumentation Laboratory, Bedford, MA, USA). Serum high-sensitivity C-reactive protein (hs-CRP) was assessed by EIA (lot no. 2410, Biometrica, Newport Beach, CA, USA).

The concentrations of interleukin 1 beta (IL-1β), interleukin-1 receptor antagonist (IL-1Ra), interleukin 2 (IL-2), interleukin 4 (IL-4), interleukin 5 (IL-5), interleukin 6 (IL-6), interleukin 7 (IL-7), interleukin 8 (IL-8), interleukin 9 (IL-9), interleukin 10 (IL-10), interleukin 12 active heterodimer (IL-12 p70), interleukin 13 (IL-13), interleukin 15 (IL-15), interleukin 17A (IL-17A), basic fibroblast growth factor (bFGF), eotaxin, granulocyte colony-stimulating factor (G-CSF), granulocyte-macrophage colony-stimulating factor (GM-CSF), interferon gamma (IFN-γ), interferon gamma-induced protein 10 (IP-10), monocyte chemoattractant protein 1 (MCP-1), macrophage inflammatory protein 1 alpha (MIP-1α), macrophage inflammatory protein 1 beta (MIP-1β), platelet-derived growth factor subunit B (PDGF-BB), regulated on activation, normal T cell expressed and secreted (RANTES), tumor necrosis factor alpha (TNF-α), and vascular endothelial growth factor (VEGF) were assessed with the use of Bio-Plex Pro™ Human Cytokine 27-plex Assay (catalog no. M500KCAF0Y, Bio-Rad Laboratories, Hercules, CA, USA). The assessed molecules were grouped (Appendix B, Table A2) according to Fundamental classification of cytokines, chemokines, and growth factors [28] and classification of growth factors [29,30].

Serum samples for assay of cytokines and growth factors were obtained from the fasting blood and stored at −80 °C until the analysis. Repeated freeze–thaw cycles were avoided. The multiplex bead array assay was performed according to manufacturer’s instructions. Serum samples were centrifuged at 10,000× *g* for 10 min at 4 °C, diluted 1:4 with Bio-Plex Sample Diluent, incubated with antibody-coupled beads, detection antibody, and streptavidin for 30 min, 30 min, and 10 min, respectively. After each coating with antigen, the plate was washed with Bio-PlexHandheld Magnetic Washer (Bio-Rad Laboratories, Hercules, CA, USA), resuspended and vortexes. Fluorescent was measured on a two-beam laser automated analyzer Bio-Plex^®^ 200 system. Data were acquired with Bio-PlexManager Software 4.0 (Bio-Rad Laboratories, Hercules, CA, USA). The values below the detection limit (OOR<) were set to zero.

The morning urine samples for the renal biomarker assay were centrifuged; the supernatants were separated and stored at −80 °C until analysis. Repeated freeze–thaw cycles were avoided. Urinary concentrations of nephrin, podocin, and WFDC2 were assessed by ELISA using commercially available kits (Cloud-Clone Corp., Wuhan, China, catalog No. SEA937Hu, SEA938Hu and SEA241Hu, respectively); the results were adjusted to urinary creatinine.

### 2.5. Statistical Analysis

Statistics 12.0 software package (Dell, Round Rock, TX, USA) was used for analysis. The outliers were excluded with Dixon’s Q test. Quantitative data are presented as medians (lower quartiles; upper quartiles); frequencies are presented as percentages (%). The Kolmogorov–Smirnov (KS) test was applied to test the normality of data distribution. Due to the fact that all of the quantitative parameters were not distributed normally, non-parametric Mann–Whitney U-test and Kruskal–Wallis H-test were used for the group comparisons. The differences in discrete parameters were assessed using the χ^2^ test. *p*-values less than 0.05 were considered as significant. Holm’s step-down procedure was applied for multiple comparisons, as recommended previously [31].

Spearman rank correlation analysis was applied to test the association between variables. Correlations with absolute value of r above 0.3 and *p*-value below 0.05 were noted.

Multiple logistic regression analysis with backward selection was used to identify predictors of different patterns of CKD in the discovery cohort. When a model was selected, we took into account the numbers of parameters included in the model, correlations between included parameters and characteristics of the model: KS statistics *p*-value and higher area under the receiver operating characteristic curve (AUC), sensitivity (Se), and specificity (Sp). We preferred the model with fewer, not correlated parameters, lower KS statistics *p*-value and higher AUC, Se, and Sp. Crude and adjusted odd ratio (OR), 95% confidence interval (95% CI), and *p*-value were calculated for parameters included in the models. The characteristics of the models (intercept, KS statistics *p*-value, Se, Sp), and the cutoff point of logistic function (L_P_) are presented. The results were checked in the validation cohort. The model was defined significant if AUC and KS statistics *p*-value in the validation cohort were comparable with those of discovery cohort and the Hosmer–Lemeshow goodness of fit (HL GOF) *p*-value were above 0.05.

## 3. Results

### 3.1. Clinical Characteristics of T2D Patients

The clinical characteristics of the patient groups are summarized in Table 1. The groups were similar by sex, age, proportion of current smokers, body mass index (BMI), waist-to-hip ratio (WHR), and diabetes duration. The prevalence of obesity, diabetic retinopathy and cardiovascular diseases did not differ significantly between the groups (all *p* > 0.05 assuming Holm’s step-down procedure). The A-CKD− group were characterized by lower proportion of the users of statins (*p* = 0.0002 vs. NA-CKD group).

The results of the renal tests and other laboratory parameters are presented in Table 2. Urinary nephrin and podocin excretions were higher in albuminuric patients (A-CKD− and A-CKD+ groups) when compared to CKD− and NA-CKD individuals (all *p* < 0.001). The CKD− and NA-CKD groups, as well as A-CKD− and A-CKD+ ones, did not differ from each other by urinary nephrin and podocin. The WFDC-2 excretion was markedly higher in men than in women (*p* < 0.000001). The NA-CKD and A-CKD+ women demonstrated elevated excretion of WFDC2 compared to CKD− women (all *p* < 0.01); these differences were not found in men.

Serum concentrations of hs-CRP were higher in patients with A-CKD+ compared to control (*p* = 0.03). This parameter demonstrated positive correlations with BMI (r = 0.34, *p* < 0.0001), HbA_1C_ (r = 0.37, *p* < 0.0001), ESR (r = 0.41, *p*< 0.0001), and plasma fibrinogen concentrations (r = 0.42, *p* < 0.0001).

### 3.2. IL-2, IL-4, IL-7, IL-9, IL-13, IL-15

**Figure 2:**
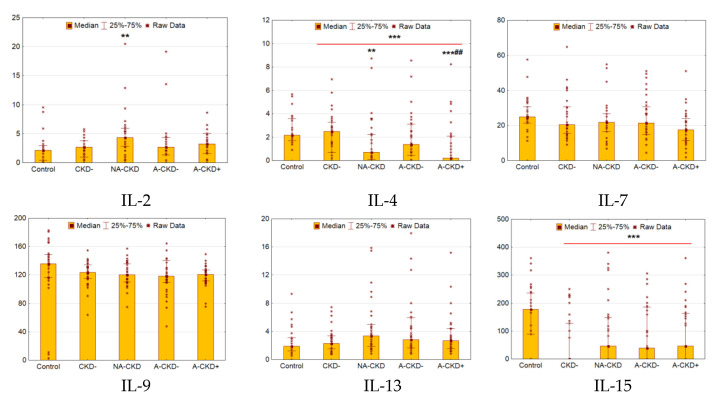
Serum concentrations of IL-2, IL-4, IL-7, IL-9, IL-13, and IL-15 (pg/mL). CKD−: patients with estimated glomerular filtration rate (eGFR) ≥60 mL/min/1.73 m^2^ and urinary albumin-to-creatinine ratio (UACR) <3.0 mg/mmol; NA-CKD: patients with eGFR <60 mL/min/1.73 m^2^ and UACR <3.0 mg/mmol; A-CKD−: patients with eGFR ≥60 mL/min/1.73 m^2^ and UACR ≥3.0 mg/mmol; A-CKD+: patients with eGFR <60 mL/min/1.73 m^2^ and UACR ≥ 3.0 mg/mmol. ** *p* < 0.01, *** *p* < 0.001 vs. non-diabetic control, ^##^ *p* < 0.01 vs. CKD−; Mann–Whitney and Kruskal–Wallis test, Holm’s step-down procedure.

Patients with T2D demonstrated decreased serum levels of IL-4 and IL-15 (*p* < 0.0001 and *p* = 0.0008 respectively, Figure 2). The NA-CKD group demonstrated markedly elevated serum IL-2 levels compared to control (*p* = 0.003). Meanwhile, the groups with declined renal function demonstrated decreased levels of IL-4 (*p* = 0.008 for NA-CKD vs. control, *p* < 0.0001 for A-CKD+ vs. control and *p* = 0.003 for A-CKD+ vs. CKD− groups). Other cytokines of this family showed no differences between diabetic groups.

### 3.3. IL-5, GM-CSF, G-CSF, IL-6, and IL-12

**Figure 3:**
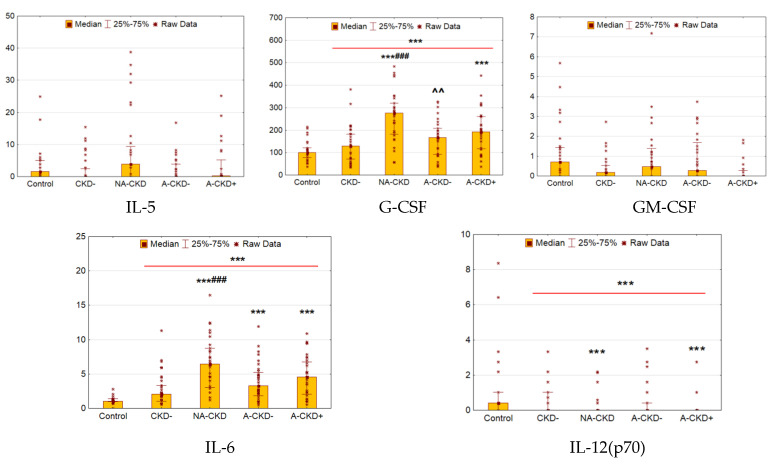
Serum concentrations of cytokines of IL-5, GM-CSF, G-CSF, IL-6, and IL-12 (pg/mL). CKD−: The group of individuals with estimated glomerular filtration rate (eGFR) ≥60 mL/min/1.73 m^2^ and urinary albumin-to-creatinine ratio (UACR) <3.0 mg/mmol; NA-CKD: Non-albuminuric chronic kidney disease, the group of individuals with eGFR <60 mL/min/1.73 m^2^ and UACR <3.0 mg/mmol; A-CKD−: Group of patients with eGFR ≥60 mL/min/1.73 m^2^ and UACR ≥3.0 mg/mmol; A-CKD+: Group of individuals with eGFR <60 mL/min/1.73 m^2^ and UACR ≥3.0 mg/mmol. *** *p* < 0.001 vs. non-diabetic control, ^###^ *p* < 0.001 vs. CKD−; ^^ *p* < 0.01 vs. NA-CKD; Mann–Whitney and Kruskal–Wallis test, Holm’s step-down procedure.

Patients with T2D were characterized by elevated serum levels of G-CSF and IL-6 when compared to control (*p* < 0.0001 and *p* = 0.0002, respectively, Figure 3), whereas concentrations of IL-12 were decreased (*p* = 0.0008).

The levels of IL-6 were increased in all groups with CKD (*p* < 0.0001for NA-CKD, A-CKD− and A-CKD+ group vs. control), the most prominent changes were found in patients with NA-CKD (*p* < 0.0001 vs. CKD− group). Patients with eGFR <60 mL/min/1.73 m^2^ (NA-CKD and A-CKD+ groups) demonstrated higher levels of G-CSF (*p* < 0.0001 and *p* = 0.0009, respectively). These groups were shown lower levels of IL-12 (*p* = 0.0009 and *p* < 0.0001 for NA-CKD and A-CKD+ groups vs. control, respectively). Additionally, patients with NA-CKD demonstrated higher levels G-CSF when compared to CKD− and A-CKD− groups (*p* < 0.0001 and *p* = 0.002 respectively).

The concentrations of IL-5 and GM-CSF demonstrated no differences between diabetic patients and the control group.

### 3.4. IL-10 and IFN-γ

**Figure 4:**
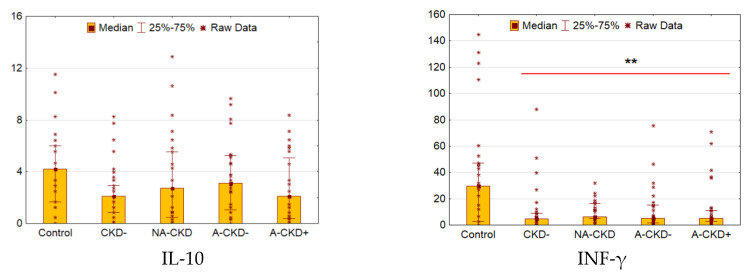
Serum concentrations of IL-10 and INF-γ (pg/mL). CKD−: The group of individuals with estimated glomerular filtration rate(eGFR) ≥60 mL/min/1.73 m^2^ and urinary albumin-to-creatinine ratio (UACR) <3.0 mg/mmol; NA-CKD: Non-albuminuric chronic kidney disease, the group of individuals with eGFR <60 mL/min/1.73 m^2^ and UACR <3.0 mg/mmol; A-CKD−: Group of patients with eGFR ≥60 mL/min/1.73 m^2^ and UACR ≥3.0 mg/mmol; A-CKD+: Group of individuals with eGFR <60 mL/min/1.73 m^2^ and UACR ≥3.0 mg/mmol. ** *p* < 0.01 vs. control; Mann–Whitney test and Kruskal–Wallis test, Holm’s step-down procedure.

There were no differences in IL-10 concentrations between control and diabetic subjects (Figure 4). The levels of INF-γ were decreased dramatically in patients with diabetes (*p* = 0.001 vs. control). No other significant differences were found between diabetic groups by these cytokines.

### 3.5. IL-1β, IL-1Ra, TNF-α, and IL-17A

**Figure 5:**
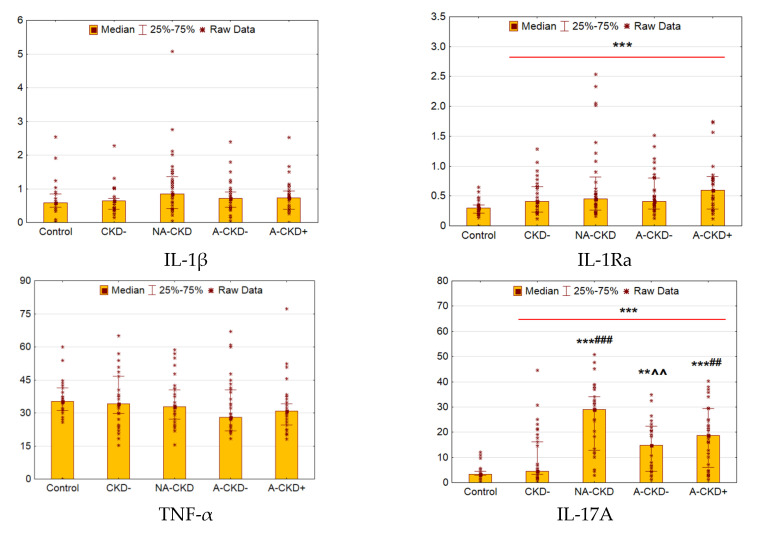
Serum concentrations of IL-1β (pg/mL), IL-1RA (ng/mL), TNF-α (pg/mL) and IL-17A (pg/mL). CKD−: The group of individuals with estimated glomerular filtration rate (eGFR) ≥60 mL/min/1.73 m^2^ and urinary albumin-to-creatinine ratio (UACR) <3.0 mg/mmol; NA-CKD: Non-albuminuric chronic kidney disease, the group of individuals with eGFR <60 mL/min/1.73 m^2^ and UACR <3.0 mg/mmol; A-CKD−: Group of patients with eGFR ≥60 mL/min/1.73 m^2^ and UACR ≥3.0 mg/mmol; A-CKD+: Group of individuals with eGFR <60 mL/min/1.73 m^2^ and UACR ≥3.0 mg/mmol. ** *p* < 0.01, *** *p* < 0.001 vs. control, ^##^ *p* < 0.01, ^###^ *p* < 0.001 vs. CKD−; ^^ *p* < 0.01 vs. NA-CKD; Mann–Whitney and Kruskal–Wallis test, Holm’s step-down procedure.

We did not reveal any differences between T2D patients and control individuals in IL-1β and TNF-α levels (Figure 5). The comparable levels of these cytokines were found in diabetic groups. The levels of IL-1Ra were elevated in 1.47 times in patients with T2D as compared to control (*p* = 0.0005).

The concentrations of IL-17A were elevated dramatically in patients with T2D when compared to control (*p* < 0.0001, Figure 5), the differences were significant for all groups with CKD (*p* < 0.0001, *p* = 0.003, and *p* < 0.0001 for NA-CKD, A-CKD− and A-CKD+ vs. control). The elevation of IL-17A levels was more prominent in groups with declined eGFR (*p* < 0.0001 for NA-CKD vs. CKD−, *p* = 0.005 for NA-CKD vs. A-CKD− and *p* = 0.004 for A-CKD+ vs. CKD−).

### 3.6. MCP-1, MIP-1α, MIP-1β, RANTES, Eotaxin, IP-10, and IL-8

**Figure 6:**
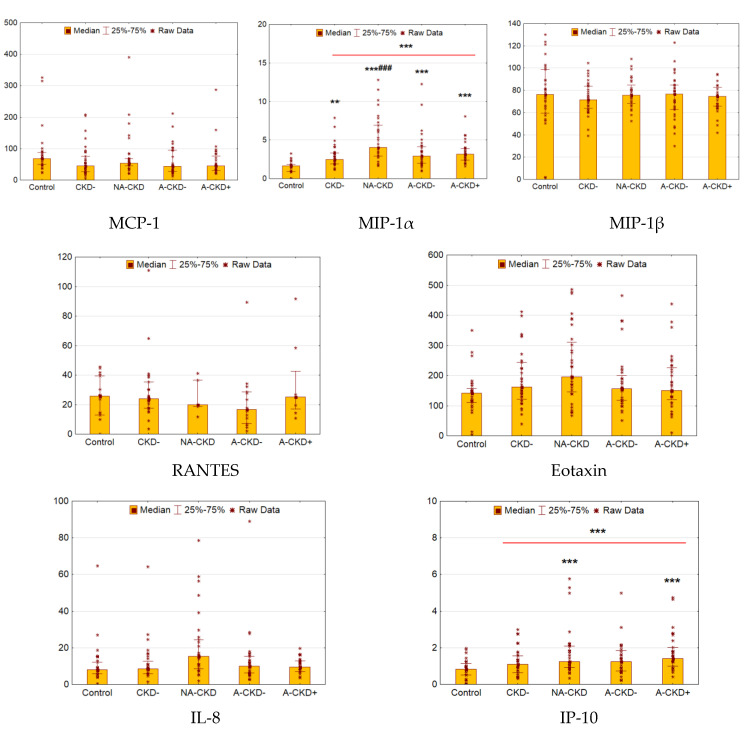
Serum concentrations of MCP-1 (CCL2), MIP-1α (CCL3), MIP-1β (CCL4), RANTES (CCL5) and eotaxin (CCL11/CCL24/CCL26) (ng/mL for RANTES and IP-10, pg/mL for others). CKD−: The group of individuals with estimated glomerular filtration rate(eGFR) ≥60 mL/min/1.73 m^2^ and urinary albumin-to-creatinine ratio(UACR) <3.0 mg/mmol; NA-CKD: Non-albuminuric chronic kidney disease, the group of individuals with eGFR <60 mL/min/1.73 m^2^ and UACR <3.0 mg/mmol; A-CKD−: Group of patients with eGFR ≥60 mL/min/1.73 m^2^ and UACR ≥3.0 mg/mmol; A-CKD+: Group of individuals with eGFR <60 mL/min/1.73 m^2^ and UACR ≥3.0 mg/mmol. ** *p* < 0.01, *** *p* < 0.001 vs. control, ^###^ *p* < 0.001 vs. CKD−; Mann–Whitney and Kruskal–Wallis test, Holm’s step-down procedure.

The levels of MIP-1α and IP-10 were elevated in patients with T2D as compared to control (*p* < 0.0001 and *p* = 0.0001, respectively, Figure 6). The levels of MCP-1, MIP-1β, RANTES, eotaxin, and IL-8 did not differ between control and diabetes subjects.

The elevation of MIP-1α was statistically significant in all diabetic groups (*p* = 0.003, *p* < 0.0001, *p* = 0.0002, and *p* < 0.0001 for CKD−, NA-CKD, A-CKD−, and A-CKD+ vs. control, respectively), demonstrating statistically significant differences between NA-CKD and CKD− patients (*p* = 0.0004). Among diabetes subjects, IP-10 was higher in the patients with eGFR <60 mL/min/1.73 m^2^ (*p* = 0.0003 for NA-CKD vs. control and *p* < 0.0001 for A-CKD+ vs. control).

### 3.7. bFGF, VEGF, and PGDF-BB

**Figure 7:**
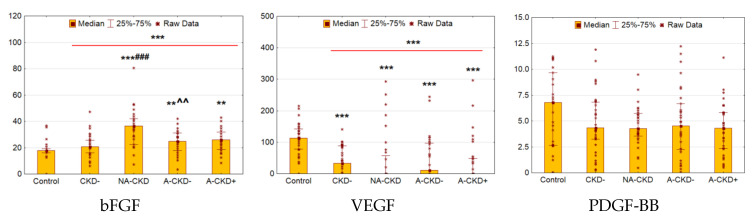
Serum concentrations of growth factors: bFGF(pg/mL), VEGF (pg/mL), and PDGF-BB (ng/mL). CKD−: The group of individuals with estimated glomerular filtration rate(eGFR) ≥60 mL/min/1.73 m^2^ and urinary albumin-to-creatinine ratio (UACR) <3.0 mg/mmol; NA-CKD: Non-albuminuric chronic kidney disease, the group of individuals with eGFR <60 mL/min/1.73 m^2^ and UACR <3.0 mg/mmol; A-CKD−: Group of patients with eGFR ≥60 mL/min/1.73 m^2^ and UACR ≥3.0 mg/mmol; A-CKD+: Group of individuals with eGFR <60 mL/min/1.73 m^2^ and UACR ≥3.0 mg/mmol. ** *p* < 0.01, *** *p* < 0.001 vs. control, ^###^ *p* < 0.001 vs. CKD−, ^^ *p* < 0.01 vs. NA-CKD; Mann–Whitney and Kruskal–Wallis test, Holm’s step-down procedure.

Serum bFGF was found to be elevated in patients with T2D compared to control (*p* < 0.0001, Figure 7). The deviation was significant for NA-CKD (*p* < 0.0001), A-CKD− (*p* = 0.002) and A-CKD+ group (*p* = 0.003). However, NA-CKD group revealed the most elevated concentrations of bFGF as compared to patients with preserved renal function (*p* < 0.0001 vs. CKD− group and *p* = 0.004 vs. A-CKD− groups).

Patients with diabetes had decreased VEGF levels (*p* < 0.0001), this decrease was significant for all diabetic groups (*p* = 0.0001 for CKD−, *p* < 0.0001 for NA-CKD, *p* = 0.0002 for A-CKD−, and *p* < 0.0001 for A-CKD+ group). The concentrations of PDGF-BB demonstrated no significant differences between diabetic and control groups.

### 3.8. Correlation Analysis and Logistic Regression Models

Diabetes duration and HbA_1C_ demonstrated no correlations with assessed cytokines and growth factors. The BMI and hs-CRP correlated positively with IL-1Ra levels (r = 0.41, *p* < 0.0001 and r = 0.36, *p*< 0.0001); meanwhile, WHRdemonstrated negative correlation with IL-4 and IL-15 (both r = −0.31, *p* = 0.02).eGFR correlated negatively with IL-6 and IL-17A (both r = −0.40, *p* < 0.0001), G-CSF (r = −0.41, *p* < 0.0001), MIP-1α (r = −0.34, *p* < 0.0001), and bFGF levels (r = −0.31, *p* = 0.0003). The excretion of WFDC2 demonstrated negative correlations with IL-10 (r = −0.31, *p* = 0.02) and VEGF (r = −0.30, *p* = 0.03). No correlations were found between cytokines, UACR, and podocyte biomarkers.

We found some correlations between assessed cytokines and growth factors. Specifically, MIP-1α demonstrated positive correlations with IL-1Ra (r = 0.35, *p* < 0.0001), IL-2 (r = 0.58, *p* < 0.0001), IL-6 (r = 0.52, *p* < 0.0001), bFGF(r = 0.51, *p* < 0.0001), and G-CSF (r = 0.68, *p* < 0.0001). The concentrations of G-CSF correlated positively with serum IL-2 (r = 0.78, *p* < 0.0001), IL-6 (r = 0.94, *p* < 0.0001), IL-17A (r = 0.92, *p* < 0.0001), bFGF(r = 0.87, *p* < 0.0001), and MIP-1α (r = 0.68, *p* < 0.0001);a correlation between G-CSF and IL-4 was negative (r = −0.37, *p* < 0.0001). The levels of bFGF demonstrated positive relationships with IL-2 (r = 0.78, *p* < 0.0001), IL-6 (r = 0.89, *p* < 0.0001), IL-17A (r = 0.89, *p* < 0.0001) and negative correlations with the levels of IL-4 (r = −0.35, *p* < 0.0001). Serum IL-17A correlated positivelywith IL-2 (r = 0.72, *p* < 0.0001)and IL-6 (r = 0.97, *p* < 0.0001); a negative correlation was found with IL-4 (r = −0.50, *p* < 0.0001). We also found positive correlations between IL-4 and IL-12 (r = 0.58, *p* < 0.0001), IL-2 and IL-6 (r = 0.73, *p* < 0.0001) and a negative correlation between IL-4 and IL-6 (r = −0.45, *p* < 0.0001).

The concentrations of IL-17A and MIP-1α, adjusted to the age and hs-CRP, were predictors for decreased eGFR in a logistic regression model (Table 3). The increase in the concentrations of IL-17A and MIP-1α by 1 pg/mL and hs-CRP by 1 mg/L elevated the riskof eGFR <60 mL/min/1.73 m^2^ by 3%, 15%, and 20%, respectively. The level of hs-CRP was significant predictor of declined eGFR after adjustment on other parameters.

The levels of eotaxin and IL-15 were included in the logistic regression model for elevated albuminuria (Table 3). The elevation of eotaxin concentration by 10 pg/mL decreased the OR of UACR ≥3.0 mg/mmol by 5%. This parameter was significant when adjusted to the concentrations of IL-15.

The elevation of IL-17A and MIP-1α concentrations increased the risk of NA-CKD by 6% and 45% per 1 pg/mL, respectively (Table 4).

Both IL-13 and HbA_1C_ demonstrated borderline statistical significance as predictors of the pattern of albuminuria with preserved renal function. After adjustment to HbA_1C_, the level of IL-13 predicted the risk of this pattern significantly. The concentrations of IL-6 and hs-CRP, adjusted to the age, were the risk factors of albuminuria with declined renal function (Table 4). The increase in levels of IL-6 and hs-CRP by 1 pg/mL and 1 mg/L, respectively, elevated A-CKD+ risk by 37% and 18% (Table 4).

## 4. Discussion

In this study, we describe the shifts in the serum panel of 27 cytokines and growth factors in patients with long-term T2D with different patterns of CKD. Depending on CKD status, included patients were classified into four groups: (1) no signs of CKD; (2) NA-CKD: eGFR <60 mL/min/1.73 m^2^ and normalalbuminuria; (3) eGFR ≥60 mL/min/1.73 m^2^ and elevated albuminuria (UACR ≥3.0 mg/mmol); (4) A-CKD: eGFR <60 mL/min/1.73 m^2^ and elevated albuminuria. For research purposes, we applied multiplex bead array assay, which allows one to simultaneously determine the concentrations of many molecules in one biological sample.

In our study, patients with T2D showed elevated levels of IL-1Ra, IL-6, IL-17A, G-CSF, IP-10, MIP-1α, and bFGF when compared to control; meanwhile, the concentrations of IL-4, IL-12, IL-15, INF-γ, and VEGF were decreased. We have found that either albuminuric or non-albuminuric patterns of CKD are associated with pro-inflammatory shifts in the panel of circulating cytokines and growth factors; the most prominent deviations were revealed in patients with reduced renal function. Among the studied molecules, IL-6, IL-17A, G-CSF, MIP-1α, and bFGF demonstrated negative correlations with eGFR; IL-17A and MIP-1α, alongside with the age and hsCRP, were associated with reduced eGFR in a multiple regression model.

Obviously, both increased production and decreased clearance may be the reasons for the increased levels of circulating cytokines in patients with CKD. Taking into account that the lowest limit of eGFR was defined as 30 mL/min × 1.73 m^2^, we do not consider the reduced clearance asa principal reason for cytokine elevation in our patients. Moreover, the cytokines, which were elevated in patients with reduced eGFR selectively (IL-1Ra, G-CSF, and IP-10), do not match the cytokines and growth factors included to EUTOX uremic toxin database (IL-1β, IL-6, IL-10, TNF-α, and VEGF) [32] or the cytokines, which can be considered as potential uremic toxins (IL-2, IL-4, IL-5, IL-12, MCP-1, and RANTES) [33]. Therefore, we believe the observed shifts in cytokine concentrations are more likely to reflect the changes in their production.

IL-6 is considered as a prototypical proinflammatory cytokine playing an important role in diabetic nephropathy [34,35]. This cytokine can be secreted by podocytes, endothelial cells, mesangial cells, and tubular epithelial cells in the kidney. Meanwhile, podocyte is the only renal cell expressing IL-6R [36]. IL-6 induces signal transducers and activators of transcription (STAT), mitogen-activated protein kinase (MAPK) and phosphoinositide 3-kinase (PI3K) [37], italso potentiates effects of angiotensin II on the kidneys [34]. In the experiment with IL-6 knockout mice, IL-6 was the key regulator for exacerbation of albuminuria and renal fibrosis [38]. Treatment with tocilizumab, an IL-6 receptor antibody, reduced proteinuria, and mesangial matrix accumulation in *db*/*db* mice, an experimental model of T2D [39].In patients with T2D, urinary IL-6 is associated with glomerular and tubular damage indicators [40].

In this study, we revealed a dramatic increase in the concentrations of IL-17A in patients with T2D and CKD, especially in those with declined renal function. A variety of cell types, including T cells, natural killers, neutrophils, macrophages, dendritic cells, lymphoid tissue inducer cells, mast cells and plasma cells, can produce IL-17A. The cytokine induces the expression of chemokines (including MCP-1), other proinflammatory cytokines (IL-6, TNF-α, and IL-1β), pro-fibrotic factors (TGF-β and fibronectin) and matrix metalloproteases [41,42]. Due to its pleiotropic character, IL-17A is involved in the development of atherosclerosis, hypertension, fibrosis, diabetic nephropathy, ischemia-reperfusion injury, glomerulonephritis, nephrotic syndrome, minimal change disease, and acute renal allograft rejection. Accordingly, inhibition of IL-17A is considered a promising target to prevent ESRD [41]. In peripheral blood of diabetic subjects with CKD, the transcript level of IL-17Ra was significantly elevated at all disease stages [43]. A significantly higher frequency of *IL17A* SNP *rs4819554 AA* homozygotes were reported among elderly individuals with eGFR <60 mL/min/1.73 m^2^, the effect was independent of the presence of T2D. Allele *rs4819554 A* had been associated to the risk of ESRD, and was also linked to the increased expression of the IL17RA protein and higher levels of Th17 cell subsets [44]. In kidney biopsy samples of T2D patients with diabetic nephropathy CD4^+^ IL-17^+^ T cells were found, and IL-17A was the key cytokine produced by these cells. The levels of IL-17A were elevated in the renal tissue and were correlated with declining eGFR. IL-17 and CD40 ligand (CD40L) synergistically enhanced IL-6, MCP-1, RANTES, TGF-β1, and NF-κB production in vitro [45]. It was found that IL-17A increases sodium–hydrogen antiporter 3 expression and sodium-chloride symporter activity in cultured proximal and distal convoluted tubule cell lines, respectively; these effects enhance sodium renal absorption and potentiate effects of the renin-angiotensin-aldosteron system on the kidney [34,46]. Contrary, it was reported that IL-17A levels in plasma and urine were reduced in patients with advanced diabetic nephropathy; administration of low-dose IL-17A prevented diabetic nephropathy in models of type 1 and type 2 diabetes [38]. The role of IL-17A in the pathogenesis of diabetic nephropathy requires further research.

The kidneys are considered as a locus of G-CSF production. Renal ischemia-reperfusion induces G-CSF gene expression in thick ascending limb cells and increased serum concentration of G-CSF [47]. The treatment with G-CSF increased a renal infiltration of myeloid-derived suppressor cells [48]. Nevertheless, G-CSF showed renal protective activity in a model of kidney ischemia-reperfusion [48] and diabetic nephropathy [49,50]. The data is inconsistent with our results, indicating an increase in the level of G-CFS in patients with reduced renal function, especially those with NA-CKD.

Some data sets indicate a role of MIP-1α in CKD. It has been demonstrated that MIP-1α recruits macrophages, lymphocytes (preferentially CD8+ T cells), and eosinophils via CCR1 or CCR5 [51,52]. The elevated renal production of MIP-1α was described in a model of adriamycin-induced tubulointerstitial fibrosis [53]. In renal biopsy specimens from patients with glomerulonephritis, glomerular CCR5-positive cells were closely correlated with extracapillary lesions and interstitial fibrosis [51]. In a model of tubulointerstitial nephritis, CCL3 and CCR5 knockout animals demonstrated better renal histology, less inflammatory infiltration, lower levels of serum creatinine, and reduced expression of KIM-1, TNF-α, and collagen in the renal tissue [54]. Knockout of CCR5 inhibited epithelial-to-mesenchymal transition [54].

In our study, bFGF (FGF-2) was increased in diabetic patients with all patterns of CKD, but not in those with normal eGFR and UACR. bFGF is considered as a key profibrotic mediator with proliferative effects on renal cells, such as interstitial fibroblasts. It was found that bFGF facilitates epithelial-to-mesenchymal transition of tubular epithelial cells [55]. Mice with fibroblast-specific disruption of the FGFR2 gene demonstrated decreased total collagen deposition, fibronectin, and alpha smooth muscle actin expression in an ischemia-reperfusion model [56,57]. On the other hand, treatment with bFGF alleviated oxidative stress and apoptosis in the kidney in *db*/*db* mice [58]. In a model of streptozotocin-induced diabetes in Sprague–Dawley rats, bFGF delayed the progression of diabetic nephropathy, possibly by inhibiting NF-κB and silencing of TGF-β1, MCP-1, IL-6, and IL-1β [59]. Perhaps this cytokine plays a dual role in the development of diabetic nephropathy.

The observed changes in the levels of IL-10 and VEGF are of interest also. These cytokines demonstrated negative correlations with urinary excretion of WFDC2, a marker of tubulointerstitial fibrosis. It was recognized that IL-10 functions as an immunosuppressive cytokine and downregulates chronic inflammatory responses [60]. In the kidney, IL-10regulatesa conversion fromclassically activated macrophages (M1) to alternatively activated ones (M2) [61]. In a model of unilateral ureteral obstruction, IL-10 knockout mice demonstrated enhanced renal fibrosis, inflammation, and severe tubular injury [62]. Treatment with IL-10 reduced macrophage infiltration, inhibited apoptosis, and decreased the fibrotic area in renal tissues in a model of obstructive nephropathy [63,64]. In patients with T2D urinary IL-10 was associated negatively with indicators of glomerular and tubular injuries [40].

The role of VEGF in CKD pathogenesis remains controversial. At early stages of the disease VEGF has been shown to promote diabetic nephropathy [65,66]. It was supposed that VEGF-induced neoangiogenesis may contribute to the initial hyperfiltration and microalbuminuria due to increased filtration area and immaturity of the neovessels respectively [67]. Besides, VEGF increases glomerular extracellular matrix deposition, disrupts the endothelial cell glycocalyx, and increases vascular permeability in rodent models of diabetes [65]. In diabetic rats, administration of anti-VEGF-A antibody attenuated albuminuria [68]. Meantime, a fall in VEGF-A promotes endothelial cell apoptosis in diabetic nephropathy and is associated with progression of CKD [65]. The loss of VEGF is related to glomerulosclerosis and tubulointerstitial fibrosis [69]. In diabetes, the reduction of podocyte numberdecreases VEGF production resulting in capillary rarefaction and deterioration of renal function [67]. In our study, we observed severe reduction of VEGF levels in T2D subjects; VEGF was a predictor of reduced eGFR.

Little is known about the role of eotaxin in diabetic kidney disease. Although patients with T2D demonstrated no significant difference in serum levels of this chemokine, the low levels of eotaxin-1 were revealed in patients with albuminuria in the logistic regression model. Eotaxin-1 is an eosinophil chemoattractant, which attracts circulating eosinophils into their niche [70,71]. A prospective cohort study among US veterans demonstrated elevated peripheral blood eosinophil count in CKD cohort compared to cohort without CKD. Among patients with T2D, eosinophilia was associated with albumin excretion [72]. Eotaxin levels were elevated in the kidneys in Long-Evans rats on a high-fat diet. These changes corresponded to increased expression of MCP-1 and the signs of glomerular and tubulointerstitial fibrosis [73]. Plasma levels of eotaxin were elevated in patients with CKD [74]. Patients with biopsy-proven diabetic nephropathy have demonstrated enhanced glomerular and tubulointerstitial production of eotaxin. These changes were more evident in those with advanced tubulointerstitial inflammation. A renal expression of eotaxin demonstrated a tendency for negative correlation with eGFR; meanwhile, there were no relationships with the levels of proteinuria [14]. These results are consistent with our findings the higher eotaxin concentrations in patients without albuminuria in the logistic regression model.

The obtained results give further support to the notion that both diabetes and CKD are associated with proinflammatory changes in the cytokine balance. A variety of factors contribute to chronic inflammatory status in diabetes, including hyperglycemia, enhanced glycemic variability, oxidative stress, obesity and adipose tissue dysfunction, and intestinal dysbiosis [75,76]. Likewise, the causes of inflammation in CKD are multifactorial and include imbalance between increased proinflammatory cytokine production (due to multiple sources of inflammatory stimuli such as oxidative stress, acidosis, volume overload, co-morbidities, especially infections) and inadequate removal (due to decreased eGFR) [57]. Therefore, in patients with diabetes and CKD the intensity of chronic inflammation can even be exacerbated. In our study, decreased renal function in T2D individuals was associated with a pro-inflammatory shift in the panel of circulating cytokines, regardless of the presence of albuminuria. Accordingly, we revealed the most prominent deviations in the patients with NA-CKD or A-CKD+. In agreement with these findings, the level of hs-CRP was significant predictor of declined eGFR.

In this study, we determined the features of the panel of circulating cytokines and growth factors in T2D patients with different patterns of CKD. We found that all patterns of CKD in patients with T2D were characterized by elevated levels of IL-6, IL-17A, and bFGF when compared to control. In multiple regression analysis, we recognized IL-17A and MIP-1α as predictors of NA-CKD. In recent years, this pattern of CKD is attracting increasing attention [3,5,77]. In T2D subjects, NA-CKD is associated with older age, female sex, better glycemic control, and increased risk of cardiovascular disease [3,4,5,77]. Although the morphological features of NA-CKD are currently poorly understood, the available data suggest that tubulointerstitial changes, interstitial fibrosis, and arteriolosclerosis can be more prominent than glomerulopathy in patients with this clinical pattern [78,79,80]. Presently, it is currently unknown whether the renal inflammation is more pronounced in this variant of CKD. The concentrations of IL-6 and hs-CRPwere found to be the risk factors of A-CKD. The role of IL-6 in DKD was discussed above. Finally, IL-13 level, adjusted to HbA_1C_, turned out to be a risk factor for albuminuria with preserved renal function.The elevated production of IL-13 in the kidney was described recently in a model of streptozotocin-induced diabetes [21]. This regulator has been identified to regulate fibrosis, exploit the IL-4Rα/Stat6 pathway, and induce chemokines MCP-1, MIP-1α, MIP-1β, and eotaxin [60]. Thus, inflammatory mediators could be potential biomarkers of various patterns of CKD in diabetes.

Currently, chronic inflammation is considered as a unifying mechanism linking diabetes, kidney pathology, and cardiovascular disease [81]; it can be an important contributor to CKD− related cardiovascular morbidity [82,83,84]. Accordingly, modification of chronic inflammation and fibrosis appears to be importantin the treatment of T2D individuals with CKD. Anti-inflammatory and anti-fibrogenic activity is an intrinsic element ofthe renal protective effect of current antihyperglycemic drugs, such as SGLT-2 inhibitors [85,86,87] and GLP-1 receptor agonists [88,89,90]. In addition, targeting specific mediators of inflammation and fibrosis is considered a promising approach to the treatment of diabetic nephropathy [9,91].

Our research is not without limitations. The cross-sectional design does not prove causality. The clinical value of the identified predictors of different CKD patterns needs to be tested in prospective studies. The study of the expression of cytokines and growth factors in the kidneys and their concentrations in urine could verify disturbances in clearance or production. The relationship between structural changes in the kidney and chronic low-grade inflammation in different CKD patterns is another matter for future research.

## 5. Conclusions

The results of this study indicate that either albuminuric or non-albuminuric patterns of CKD are associated with pro-inflammatory shifts in the panel of circulating cytokines and growth factors in T2D patients. In these patients, serum levels of IL-6, IL-17A, G-CSF, MIP-1α, and bFGF demonstrate negative relationships with eGFR, but not with albuminuria or urinary podocyte biomarkers; IL-10 and VEGF correlate negatively with WFDC2, a marker of tubulointerstitial fibrosis. The shifts in the cytokine panel are differently associated with the patterns of CKD: IL-17A and MIP-1α are predictors of non-albuminuric CKD, IL-13 is associated with the pattern of albuminuria and preserved renal function; meanwhile, IL-6 and hs-CRP could be predictors of albuminuria with eGFR decline.

## Figures and Tables

**Table 1:** Clinical characteristics of T2D patients with albuminuricand non-albumiuric CKD patterns

Parameter	CKD−	NA-CKD	A-CKD−	A-CKD+
N	33	33	32	32
Sex, M/F, *n*	12/21	12/21	12/20	12/20
Age, years	64 (58; 69)	65 (58; 67)	63 (57; 67)	65 (58; 69.5)
Smokers, *n* (%)	3 (9.1%)	5 (15.1%)	4 (12.5%)	3 (9.4%)
BMI, kg/m^2^	34.4 (28.1; 36.9)	34.7 (30.1; 38.9)	33.8 (31.2; 40.0)	32.9 (30.3; 36.7)
WHR	0.97 (0.93; 1.1)	1.02 (0.9; 1.1)	1.00 (0.93; 1.1)	1.00 (0.91; 1.07)
Diabetes duration, years	13 (11; 16)	14 (12; 19)	13.5 (11; 17)	13.5 (11; 17.5)
*Diabetic complications and associated diseases*				
Obesity, *n* (%)	21 (63.6%)	24 (72.7%)	25 (78.2%)	26 (81.3%)
Diabetic retinopathy, *n* (%)	17 (51.5%)	17 (51.5%)	20 (62.5%)	21 (65.6%)
Arterial hypertension, *n* (%)	31 (93.9%)	33 (100%)	31 (96.9%)	32 (100%)
Coronary artery disease, *n* (%)	13 (39.4%)	16 (48.5%)	16 (50.0%)	18 (56.2%)
Myocardial infarction in anamnesis, *n* (%)	4 (12.1)	7 (21.2%)	5 (15.6%)	10 (31.3%)
Cerebrovascular event in anamnesis, *n* (%)	1 (3.0%)	6 (18.2%)	3 (9.4%)	2 (6.3%)
Peripheral artery disease, *n* (%)	17 (51.5%)	22 (66.7%)	20 (62.5%)	23 (71.9%)
*Treatment*				
Metformin, *n* (%)	26 (78.8%)	20 (60.6%)	23 (71.9%)	20 (62.5%)
Sulfonylurea, *n* (%)	8 (24.2%)	15 (45.5%)	12 (37.5%)	9 (28.1%)
Insulin, *n* (%)	21 (63.6%)	22 (66.7%)	21 (65.6%)	26 (81.3%)
RAS blockers, *n* (%)	33 (100%)	27 (81.8%)	26 (81.3%)	27 (84.4%)
Diuretics, *n* (%)	12 (36.4%)	17 (51.5%)	16 (50.0%)	17 (53.1%)
Calcium channel blockers, *n* (%)	8 (24.2%)	16 (48.5%)	10 (31.3%)	14 (43.8%)
Antiplatelet agents, *n* (%)	21 (63.6%)	27 (81.8%)	19 (59.4%)	24 (75.0%)
Statins, *n* (%)	13 (39.4%)	23 (69.7%)	7 (21.9%) ^###^	17 (53.1%)

Continuous data are presented as medians (lower quartile; upper quartile), frequencies are presented as number of patient (percentage in a group), ^###^ *p* <0.001 vs. NA-CKD, Kruskal–Wallis and Mann–Whitney test, Holm’s step-down procedure. CKD−: The group of individuals with estimated glomerular filtration rate (eGFR) ≥60 mL/min/1.73 m^2^ and urinary albumin-to-creatinine ratio (UACR) <3.0 mg/mmol; NA-CKD: Non-albuminuric chronic kidney disease, the group of individuals with eGFR< 60 mL/min/1.73 m^2^ and UACR <3.0 mg/mmol; A-CKD−: Group of patients with eGFR ≥60 mL/min/1.73 m^2^ and UACR ≥3.0 mg/mmol; A-CKD+: Group of individuals with eGFR <60 mL/min/1.73 m^2^ and UACR ≥3.0 mg/mmol.

**Table 2:** Laboratory parameters in T2D patients with albuminuric and non-albuminuric CKD

Parameter		CKD−	NA-CKD	A-CKD−	A-CKD+
**N**		**33**	**33**	**32**	**32**
*Renal tests*					
Serum creatinine, μmol/L		76 (68; 87)	111 (102; 124) ***	87 (79; 97) ^§§§ ###^	117 (98; 144) ***
eGFR, mL/min/1.73 m^2^		84 (73; 94)	51 (45; 55) ***	71 (65; 77) ^§§§ ###^	51 (43; 55) ***
UACR, mg/mmol		0.6 (0.3; 0.9)	0.6 (0.3; 0.9)	13.5 (6.4; 38.4) *** ^###^	14.1 (7.2; 82.5) *** ^###^
Urinary nephrin excretion, ng/mmol		12.1 (4.7; 21.1)	7.8 (4.1; 16.3)	22.6 (16.6; 36.2) *** ^###^	22.2 (17.1; 32.9) *** ^###^
Urinary podocin excretion, ng/mmol		133 (86; 222)	105 (65.4; 193.9)	250 (144; 421) *** ^###^	268 (134; 411) *** ^###^
Urinary excretion of WFDC2, ng/mmol	M	442 (310; 941) ^+++^	723 (406; 811) ^+++^	N/D	628 (410; 1277) ^+++^
	F	5.6 (0; 81)	129 (0; 330) **	N/D	231 (110; 619) **
*Other biochemical parameters*					
HbA_1C_	%	8.48 (7.5; 9.8)	9.06 (7.9; 10.26)	9.36 (8.25; 11.58)	8.81 (7.64; 10.1)
	mmol/mol	69 (58; 84)	76 (63; 89)	79 (67; 103)	73 (60; 87)
Total cholesterol, mmol/L		4.92 (4.32; 5.51)	5.14 (4.11; 6.07)	4.89 (4.06; 5.89)	5.5 (4.19; 6.25)
LDL-cholesterol, mmol/L		3.02 (2.7; 3.59)	3.21 (2.51; 3.92)	3.19 (2.6; 3.95)	3.26 (2.54; 4.18)
HDL-cholesterol, mmol/L		1.13 (1; 1.31)	1.22 (1.09; 1.41)	1.07 (0.93; 1.25) ^#^	1.12 (1; 1.41)
Triglycerides, mmol/L		1.76 (1.15; 2.47)	1.9 (1.5; 2.6)	2.2 (1.64; 3.3)	1.93 (1.54; 3.08)
Uric acid, μmol/L		298 (243; 352)	341 (315; 392)	322 (281; 383)	366 (272; 418)
Serum hs-CRP, mg/L		4.1 (2.3; 8.2)	6.2 (2.3; 8.4)	4.4 (1.4; 7.3)	6.7 (2.2; 13.1)
*Hematology and coagulation tests*					
Hemoglobin, g/L		139 (131; 148)	141 (128; 151)	141 (127; 152)	141 (126; 153)
RBC, × 1012/L		4.79 (4.5; 4.9)	4.72 (4.44; 5.06)	4.85 (4.6; 5.13)	4.7 (4.26; 4.87)
WBC, × 109/L		6.18 (5.93; 7.65)	7.44 (5.61; 8.34)	7.52 (6.03; 8.85)	7.31 (5.53; 8.15)
Platelets, × 109/L		244 (218; 269)	253 (207; 282)	247 (212; 278)	206 (181; 275)
ESR, mm/h		15 (9; 22)	19 (12; 26.5)	22.5 (15.5; 29)	23.5 (18; 30) **
Fibrinogen, g/L		4.15 (3.3; 5.3)	3.9 (3.2; 4.45)	4.4 (3.7; 5.5)	4.6 (4; 5.3)
SFMCs, mg/dL		4 (3.5; 13.5)	14 (3.5; 21)	10.5 (6; 21.5)	12 (8; 19)
D-dimer, ng/mL		255 (215; 309)	251 (231; 385)	268 (231; 297)	277 (238; 327)

Continues data are presented as medians (lower quartile; upper quartile), ** *p* < 0.01, *** *p* < 0.001 vs. CKD−, ^#^ *p* < 0.05, ^###^ *p* < 0.001 vs. NA-CKD, ^§§§^ *p* < 0.001 vs. A-CKD+, ^+++^*p* < 0.001 vs. female patients with T2D; Mann–Whitney and Kruskal–Wallis test, Holm’s step-down procedure. CKD−: The group of individuals with estimated glomerular filtration rate (eGFR) ≥60 mL/min/1.73 m^2^ and urinary albumin-to-creatinine ratio (UACR) <3.0 mg/mmol; NA-CKD: Non-albuminuric chronic kidney disease, the group of individuals with eGFR <60 mL/min/1.73 m^2^ and UACR <3.0 mg/mmol; A-CKD−: Group of patients with eGFR ≥60 mL/min/1.73 m^2^ and UACR ≥3.0 mg/mmol; A-CKD+: Group of individuals with eGFR <60 mL/min/1.73 m^2^ and UACR ≥3.0 mg/mmol; hs-CRP, high-sensitively C-reactive protein; WFDC2, whey acidic protein four-disulfide core domain protein 2.

**Table 3:** Predictors for declined eGFR and elevated UACR in T2D patients

Parameter	Crude OR (95% CI), *p*-Value	Adjusted OR (95% CI), *p*-Value
*eGFR <60 mL/min × 1.73 m^2^* ^1^		
IL-17A, pg/mL	1.04(1.01–1.09), *p* = 0.004	1.03(1.01–1.09), *p* = 0.01
MIP-1α, pg/mL	1.30(1.06–1.49), *p* = 0.02	1.15(1.02–1.50), *p* = 0.03
Serum hs-CRP, mg/L	1.01(0.96–1.05), *p* = 0.10	1.20(1.02–1.30), *p* = 0.02
Age, years	1.06 (0.98–1.15), *p* = 0.16	1.04(0.99–1.14), *p* = 0.08
*UACR ≥3.0 mg/mmol* ^2^		
Eotaxin, 10 pg/mL	0.98(0.94–1.00), *p* = 0.09	0.95(0.90–1.00), *p* = 0.03
IL-15, 10 pg/mL	1.03(0.98–1.06), *p* = 0.20	1.04(0.98–1.07), *p* = 0.09

The multiple logistic regression models of declined eGFR (1) and elevaled UACR (2); ^1^ The characteristics of the model 1: Intercept = −5.56, KS *p*-value < 0.0001, AUC = 0.78, Se = 0.72, Sp = 0.73, L_P_ = 0.48, HL GOF *p*-value = 0.57; ^2^ The characteristics of the model 2: Intercept = 0.516, KS *p*-value = 0.02, AUC = 0.68, Se = 0.64, Sp = 0.62 for L_P_ = 0.50, HL GOF *p*-value = 0.12.CKD−: The group of individuals with estimated glomerular filtration rate (eGFR) ≥60 mL/min/1.73 m^2^ and urinary albumin-to-creatinine ratio (UACR) <3.0 mg/mmol; NA-CKD: Non-albuminuric chronic kidney disease, the group of individuals with eGFR <60 mL/min/1.73 m^2^ and UACR <3.0 mg/mmol; A-CKD−: Group of patients with eGFR ≥60 mL/min/1.73 m^2^ and UACR ≥3.0 mg/mmol; A-CKD+: Group of individuals with eGFR <60 mL/min/1.73 m^2^ and UACR ≥3.0 mg/mmol.

**Table 4:** Predictors of different CKD patterns in T2D patients

Parameter	Crude OR (95% CI), *p*-Value	Adjusted OR (95% CI), *p*-Value
*NA-CKD* ^1^		
IL-17A, pg/mL	1.08 (1.04–1.18), *p* = 0.001	1.06 (1.02–1.12), *p* = 0.004
MIP-1α, pg/mL	1.70 (1.20–2.30), *p* = 0.008	1.45 (1.02–2.06), *p* = 0.03
*A-CKD−* ^2^		
IL-13, pg/mL	1.20 (0.96–1.50), *p* = 0.09	1.24 (1.01–1.54), *p* = 0.04
HbA_1C_, %	1.15 (0.94–1.52), *p* = 0.12	1.30 (0.98–1.62), *p* = 0.06
*A-CKD+* ^3^		
IL-6, pg/mL	1.27 (1.02–1.64), *p* = 0.02	1.37 (1.08–1.69), *p* = 0.009
Serum hs-CRP, mg/L	1.06 (0.92–1.32), *p* = 0.21	1.18 (1.01–1.36), *p* = 0.04
Age, years	1.03 (0.90; 1.12), *p* = 0.26	1.09 (0.98–1.20), *p* = 0.10

The multiple logistic regression models of CKD in patients with T2D. ^1^ The characteristics of the model for NA-CKD: Intercept = −2.84, KS *p*-value = 0.0004, AUC = 0.84, Se = 0.76, Sp = 0.74, L_P_ = 0.48, HL GOF *p*-value = 0.36; ^2^ The characteristics of the model for A-CKD−: Intercept = −3.12, KS *p*-value = 0.04, AUC = 0.72, Se = 0.68, Sp = 0.64, L_P_ = 0.46, HL GOF *p*-value = 0.12; ^3^ The characteristics of the model for A-CKD+: Intercept = −4.70, KS *p*-value = 0.003, AUC = 0.82, Se = 0.76, Sp = 0.80, L_P_ = 0.51, HL GOF *p*-value = 0.88. CKD−: The group of individuals with estimated glomerular filtration rate (eGFR) ≥60 mL/min/1.73 m^2^ and urinary albumin-to-creatinine ratio (UACR) <3.0 mg/mmol; NA-CKD: Non-albuminuric chronic kidney disease, the group of individuals with eGFR <60 mL/min/1.73 m^2^ and UACR <3.0 mg/mmol; A-CKD−: Group of patients with eGFR ≥60 mL/min/1.73 m^2^ and UACR ≥3.0 mg/mmol; A-CKD+: Group of individuals with eGFR <60 mL/min/1.73 m^2^ and UACR ≥3.0 mg/mmol.

## Appendix A

**Table A1:** Characteristics of T2D patients in the discovery and validation cohorts

Parameter	Discovery Cohort	Validation Cohort
N	86	44
Sex, M/F, *n* (%)	29/57 (33.7%/66.3%)	16/28 (36.4%/63.4%)
Age, years	65 (58; 69)	65.5 (58.5; 70)
Smokers, *n* (%)	10 (11.6%)	5 (11.4%)
BMI, kg/m^2^	33.4 (29.4; 37.4)	33.2 (29.8; 38.9)
Diabetes duration, years	13.5 (11; 17)	14 (11; 18)
CKD−/NA-CKD/A-CKD−/A-CKD+, *n*	22/22/21/21	11/11/11/11

Continuous data are presented as medians (lower quartile; upper quartile), frequencies are presented as number of patient (percentage in a group). BMI: Body mass index; CKD−: The group of individuals with estimated glomerular filtration rate (eGFR) ≥60 mL/min/1.73 m^2^ and urinary albumin-to-creatinine ratio (UACR) <3.0 mg/mmol; NA-CKD: Non-albuminuric chronic kidney disease, the group of individuals with eGFR <60 mL/min/1.73 m^2^ and UACR <3.0 mg/mmol; A-CKD−: Group of patients with eGFR ≥60 mL/min/1.73 m^2^ and UACR ≥3.0 mg/mmol; A-CKD+: Group of individuals with eGFR <60 mL/min/1.73 m^2^ and UACR ≥3.0 mg/mmol.

## Appendix B

**Table A2:** Panel of cytokines and growth factors assessed in the study

Family	Protein
Archetypical cytokines signaling through classical-cytokine receptors *Type I helical Cytokine families signaling through Class I cytokine receptors* *(CRF1 family or Hematopoietin family)*	
IL-2 Family, or Common Gamma Chain Receptor Family	IL-2, IL-4 subfamily(IL-4, IL-13), IL-9, IL-15, and IL-7 subfamily(IL-7)
Common Beta Chain Receptor Cytokine Family	IL-5, Colony Stimulating Factor 2/Granulocyte Monocyte-Stimulating Factor (CSF2/GM-CSF)
Prolactin family	Colony Stimulating Factor 3/Granulocyte-Stimulating Factor (CSF3/G-CSF)
IL-6 Family	IL-6
IL-12 Family	IL-12 (p70)
*Type II Cytokine families signaling through Class II cytokine receptors* *(CRF2 family or IL-10/IFN superfamily)*	
IL-10 Family	IL-10
Type II IFN	IFNγ
**Cytokine families signaling through immunoglobulin** (**Ig**) **superfamily cytokine receptors** ***non-Receptor tyrosine-kinase** (**RTK**)*	
IL-1 Family	IL-1β, IL-1Ra
**Cytokine TNF family signaling through TNF receptor family**	
Family A	TNFα (TNFSF2)
**Chemokine superfamily signaling through chemokine receptors** (**seven-transmembrane heptahelical**(**serpentine**) **receptors associated with G-protein trimeric system**)	
Chemokine CC Motif Ligand Family (CCL)	MCP1 (CCL2), MIP-1α (CCL3), MIP-1β (CCL4), RANTES (CCL5) and eotaxin(CCL11)
Chemokine CXC Motif Ligand Family (CXCL)	IP-10 (CXCL10), IL-8 (CXCL8)
**Orphan and other cytokine family members**	
IL-17 Family	IL-17A
**Growth factors and signaling proteins**	
Platelet-Derived Growth Factor Family	PDGF-BB (Platelet-Derived Growth Factor subunit B)
Vascular Endothelial Growth Factor Family	VEGF (Vascular Endothelial Growth Factor)/Vascular Permeability Factor (VPF)
Fibroblast Growth Factor Family	bFGF(FGF2/FGF-β)

The assessed cytokines and growth factors were grouped according to Fundamental classification of cytokines, chemokines, and growth factors [28] and classification of growth factors [29,30].

## References

[1] Bethesda: National Institutes of Health, National Institute of Diabetes and Digestive and Kidney Diseases (2018). 2018 USRDS Annual Data Report: Epidemiology of Kidney Disease in the United States. https://www.usrds.org/2018/view/Default.aspx.

[2] International Diabetes Federation (2019). IDF Diabetes Atlas.

[3] Viazzi F., Russo G.T., Ceriello A., Fioretto P., Giorda C., De Cosmo S., Pontremoli R. (2019). Natural history and risk factors for diabetic kidney disease in patients with T2D: Lessons from the AMD-annals. J. Nephrol..

[4] Korbut A.I., Klimontov V.V., Vinogradov I.V., Romanov V.V. (2019). Risk factors and urinary biomarkers of non-albuminuric and albuminuric chronic kidney disease in patients with type 2 diabetes. World J. Diabetes.

[5] Pugliese G., Solini A., Bonora E., Fondelli C., Orsi E., Nicolucci A., Penno G., RIACE Study Group (2014). Chronic kidney disease in type 2 diabetes: Lessons from the Renal Insufficiency and Cardiovascular Events (RIACE) Italian Multicentre Study. Nutr. Metab. Cardiovasc. Dis..

[6] Di Vincenzo A., Bettini S., Russo L., Mazzocut S., Mauer M., Fioretto P. (2020). Renal structure in type 2 diabetes: Facts and misconceptions [published online ahead of print, 2020 Jul 12]. J. Nephrol..

[7] Donate-Correa J., Martín-Núñez E., Muros-de-Fuentes M., Mora-Fernández C., Navarro-González J.F. (2015). Inflammatory cytokines in diabetic nephropathy. J. Diabetes Res..

[8] Pérez-Morales R.E., Del Pino M.D., Valdivielso J.M., Ortiz A., Mora-Fernández C., Navarro-González J.F. (2019). Inflammation in diabetic kidney disease. Nephron.

[9] Rayego-Mateos S., Morgado-Pascual J.L., Opazo-Ríos L., Guerrero-Hue M., García-Caballero C., Vázquez-Carballo C., Mas S., Sanz A.B., Herencia C., Mezzano S. (2020). Pathogenic pathways and therapeutic approaches targeting inflammation in diabetic nephropathy. Int. J. Mol. Sci..

[10] Kelly K.J., Dominguez J.H. (2010). Rapid progression of diabetic nephropathy is linked to inflammation and episodes of acute renal failure. Am. J. Nephrol..

[11] Klessens C.Q.F., Zandbergen M., Wolterbeek R., Bruijn J.A., Rabelink T.J., Bajema I.M., IJpelaar D.H.T. (2017). Macrophages in diabetic nephropathy in patients with type 2 diabetes. Nephrol. Dial. Transplant..

[12] Zhang X., Yang Y., Zhao Y. (2019). Macrophage phenotype and its relationship with renal function in human diabetic nephropathy. PLoS ONE.

[13] Wu C.C., Chen J.S., Lu K.C., Chen C.C., Lin S.H., Chu P., Sytwu H.K., Lin Y.F. (2010). Aberrant cytokines/chemokines production correlate with proteinuria in patients with overt diabetic nephropathy. Clin. Chim. Acta.

[14] Araújo L.S., Torquato B.G.S., da Silva C.A., Dos Reis Monteiro M.L.G., Dos Santos Martins A.L.M., da Silva M.V., Dos Reis M.A., Machado J.R. (2020). Renal expression of cytokines and chemokines in diabetic nephropathy. BMC Nephrol..

[15] Salti T., Khazim K., Haddad R., Campisi-Pinto S., Bar-Sela G., Cohen I. (2020). Glucose induces IL-1α-dependent inflammation and extracellular matrix proteins expression and deposition in renal tubular epithelial cells in diabetic kidney disease. Front. Immunol..

[16] Stefan G., Stancu S., Zugravu A., Petre N., Mandache E., Mircescu G. (2019). Histologic predictors of renal outcome in diabetic nephropathy: Beyond renal pathology society classification. Medicine.

[17] Konenkov V.I., Klimontov V.V., Myakina N.E., Tyan N.V., Fazullina O.N., Romanov V.V. (2015). Increased serum concentrations of inflammatory cytokines in type 2 diabetic patients with chronic kidney disease. Ther. Arch..

[18] Araújo L.S., da Silva M.V., da Silva C.A., Borges M.F., Palhares H.M.D.C., Rocha L.P., Corrêa R.R.M., Rodrigues Júnior V., Dos Reis M.A., Machado J.R. (2020). Analysis of serum inflammatory mediators in type 2 diabetic patients and their influence on renal function. PLoS ONE.

[19] Niewczas M.A., Pavkov M.E., Skupien J., Smiles A., Dom Z.I., Wilson J.M., Park J., Nair V., Schlafly A., Saulnier P.J. (2019). A signature of circulating inflammatory proteins and development of end-stage renal disease in diabetes. Nat. Med..

[20] Rea I.M., Gibson D.S., McGilligan V., McNerlan S.E., Alexander H.D., Ross O.A. (2018). Age and age-related diseases: Role of inflammation triggers and cytokines. Front. Immunol..

[21] Sierra-Mondragon E., Molina-Jijon E., Namorado-Tonix C., Rodríguez-Muñoz R., Pedraza-Chaverri J., Reyes J.L. (2018). All-trans retinoic acid ameliorates inflammatory response mediated by TLR4/NF-κB during initiation of diabetic nephropathy. J. Nutr. Biochem..

[22] Zhang Y., Li X., Di Y.P. (2020). Fast and efficient measurement of clinical and biological samples using immunoassay-based multiplexing systems. Methods Mol. Biol..

[23] Lioudaki E., Stylianou K.G., Petrakis I., Kokologiannakis G., Passam A., Mikhailidis D.P., Daphnis E.K., Ganotakis E.S. (2015). Increased urinary excretion of podocyte markers in normoalbuminuric patients with diabetes. Nephron.

[24] Wada Y., Abe M., Moritani H., Mitori H., Kondo M., Tanaka-Amino K., Eguchi M., Imasato A., Inoki Y., Kajiyama H. (2016). Potential of urinary nephrin as a biomarker reflecting podocyte dysfunction in various kidney disease models. Exp. Biol. Med..

[25] El-Shazly A.A.A., Sallam A.M., El-Hefnawy M.H., El-Mesallamy H.O. (2019). Epidermal growth factor receptor and podocin predict nephropathy progression in type 2 diabetic patients through interaction with the autophagy influencer ULK-1. J. Diabetes Complicat..

[26] Wan J., Wang Y., Cai G., Liang J., Yue C., Wang F., Song J., Wang J., Liu M., Luo J. (2016). Elevated serum concentrations of HE4 as a novel biomarker of disease severity and renal fibrosis in kidney disease. Oncotarget.

[27] Chen P., Yang Q., Li X., Qin Y. (2017). Potential association between elevated serum human epididymis protein 4 and renal fibrosis: A systemic review and meta-analysis. Medicine.

[28] García Morán G.A., Parra-Medina R., Cardona A.G., Cardona A.G., Quintero-Ronderos P., Rodríguez É.G., Anaya J.M., Shoenfeld Y., Rojas-Villarraga A. (2013). Cytokines, chemokines and growth factors. Autoimmunity: From Bench to Bedside.

[29] Bafico A., Aaronson S.A., Kufe D.W., Pollock R.E., Weichselbaum R.R. (2003). Classification of growth factors and their receptors. Holland-Frei Cancer Medicine.

[30] Sherbet G.V., Gajanan V. (2011). Growth Factors and Their Receptors in Cell Differentiation, Cancer and Cancer Therapy.

[31] Pfister I.B., Zandi S., Gerhardt C., Spindler J., Reichen N., Garweg J.G. (2020). Risks and Challenges in Interpreting Simultaneous Analyses of Multiple Cytokines. Transl. Vis. Sci. Technol..

[32] EUTOX Uremic Toxin Database. https://www.uremic-toxins.org/eutox-database/.

[33] Castillo-Rodríguez E., Pizarro-Sánchez S., Sanz A.B., Ramos A.M., Sanchez-Niño M.D., Martin-Cleary C., Fernandez-Fernandez B., Ortiz A. (2017). Inflammatory Cytokines as Uremic Toxins: “Ni Son Todos Los Que Estan, Ni EstanTodos Los Que Son”. Toxins.

[34] Norlander A.E., Madhur M.S. (2017). Inflammatory cytokines regulate renal sodium transporters: How, where, and why?. Am. J. Physiol.-Ren. Physiol..

[35] Feigerlová E., Battaglia-Hsu S.F. (2017). IL-6 signaling in diabetic nephropathy: From pathophysiology to therapeutic perspectives. Cytokine Growth Factor Rev..

[36] Su H., Lei C.T., Zhang C. (2017). Interleukin-6 signaling pathway and its role in kidney disease: An update. Front. Immunol..

[37] Magno A.L., Heart L.Y., Carnagarin R., Schlaich M.P., Matthews V.B. (2019). Current knowledge of IL-6 cytokine family members in acute and chronic kidney disease. Biomedicines.

[38] Mohamed R., Jayakumar C., Chen F., Enciu A.M., Albulescu L., Necula L.G., Mambet C., Anton G., Tanase C. (2016). Low-dose IL-17 therapy prevents and reverses diabetic nephropathy, metabolic syndrome, and associated organ fibrosis. J. Am. Soc. Nephrol..

[39] Wu R., Liu X., Yin J., Wu H., Cai X., Wang N., Qian Y., Wang F. (2018). IL-6 receptor blockade ameliorates diabetic nephropathy via inhibiting inflammasome in mice. Metabolism.

[40] Sangoi M.B., Carvalho J.A.M., Guarda N.S., Duarte T., Duarte M.M.M.F., Premaor M.O., Comim F.V., Moretto M.B., Moresco R.N. (2019). Association between urinary levels of interleukin-6, interleukin-10 and tumor necrosis factor-alpha with glomerular and tubular damage indicators in patients with type 2 diabetes. Clin. Lab..

[41] Cortvrindt C., Speeckaert R., Moerman A., Delanghe J.R., Speeckaert M.M. (2017). The role of interleukin-17A in the pathogenesis of kidney diseases. Pathology.

[42] Ma J., Li Y.J., Chen X., Kwan T., Chadban S.J., Wu H. (2019). Interleukin 17A promotes diabetic kidney injury. Sci. Rep..

[43] Perlman A.S., Chevalier J.M., Wilkinson P., Liu H., Parker T., Levine D.M., Sloan B.J., Gong A., Sherman R., Farrell F.X. (2015). Serum inflammatory and immune mediators are elevated in early stage diabetic nephropathy. Ann. Clin. Lab. Sci..

[44] Coto E., Gómez J., Suárez B., Tranche S., Díaz-Corte C., Ortiz A., Ruiz-Ortega M., Coto-Segura P., Batalla A., López-Larrea C. (2015). Association between the IL17RA rs4819554 polymorphism and reduced renal filtration rate in the Spanish RENASTUR cohort. Hum. Immunol..

[45] Kuo H.L., Huang C.C., Lin T.Y., Lin C.Y. (2018). IL-17 and CD40 ligand synergistically stimulate the chronicity of diabetic nephropathy. Nephrol. Dial. Transplant..

[46] Norlander A.E., Saleh M.A., Kamat N.V., Ko B., Gnecco J., Zhu L., Dale B.L., Iwakura Y., Hoover R.S., McDonough A.A. (2016). Interleukin-17A regulates renal sodium transporters and renal injury in angiotensin II-induced hypertension. Hypertension.

[47] Nishida M., Hamaoka K. (2006). How does G-CSF act on the kidney during acute tubular injury?. Nephron Exp. Nephrol..

[48] Yan J.J., Ryu J.H., Piao H., Hwang J.H., Han D., Lee S.K., Jang J.Y., Lee J., Koo T.Y., Yang J. (2020). Granulocyte colony-stimulating factor attenuates renal ischemia-reperfusion injury by inducing myeloid-derived suppressor cells. J. Am. Soc. Nephrol..

[49] So B.I., Song Y.S., Fang C.H., Park J.Y., Lee Y., Shin J.H., Kim H., Kim K.S. (2013). G-CSF prevents progression of diabetic nephropathy in rat. PLoS ONE.

[50] Erbas O., Yapislar H., Oltulu F., Yavasoğlu A., Aktug H., Taskiran D. (2014). Nephro-protective effect of granulocyte colony-stimulating factor in streptozotocin induced diabetic rats. Biotech. Histochem..

[51] Ruster C., Wolf G. (2008). The role of chemokines and chemokine receptors in diabetic nephropathy. Front. Biosci..

[52] Bhavsar I., Miller C.S., Al-Sabbagh M., Preedy V., Patel V. (2015). Macrophage inflammatory protein-1 alpha (MIP-1 alpha)/CCL3: As a biomarker. General Methods in Biomarker Research and Their Applications.

[53] Zheng G., Wang Y., Mahajan D., Qin X., Wang Y., Wang Y., Alexander S.I., Harris D.C. (2005). The role of tubulointerstitial inflammation. Kidney Int..

[54] Correa-Costa M., Braga T.T., Felizardo R.J., Andrade-Oliveira V., Perez K.R., Cuccovia I.M., Hiyane M.I., da Silva J.S., Câmara N.O. (2014). Macrophage trafficking as key mediator of adenine-induced kidney injury. Mediat. Inflamm..

[55] Strutz F. (2009). The role of FGF-2 in renal fibrogenesis. Front. Biosci..

[56] Xu Z., Dai C. (2017). Ablation of FGFR2 in fibroblasts ameliorates kidney fibrosis after ischemia/reperfusion injury in mice. Kidney Dis..

[57] Dai L., Golembiewska E., Lindholm B., Stenvinkel P. (2017). End-stage renal disease, inflammation and cardiovascular outcomes. Expanded Hemodialysis—Innovative Clinical Approach in Dialysis. Contrib Nephrol..

[58] Wei T., Shu Q., Ning J., Wang S., Li C., Zhao L., Zheng H., Gao H. (2020). The protective effect of basic fibroblast growth factor on diabetic nephropathy through remodeling metabolic phenotype and suppressing oxidative stress in mice. Front. Pharmacol..

[59] Sheng W.S., Xu H.L., Zheng L., Zhuang Y.D., Jiao L.Z., Zhou J.F., ZhuGe D.L., Chi T.T., Zhao Y.Z., Lan L. (2018). Intrarenal delivery of bFGF-loaded liposome under guiding of ultrasound-targeted microbubble destruction prevent diabetic nephropathy through inhibition of inflammation. Artif. Cells Nanomed. Biotechnol..

[60] Wynn T.A. (2008). Cellular and molecular mechanisms of fibrosis. J. Pathol..

[61] Landis R.C., Quimby K.R., Greenidge A.R. (2018). M1/M2 macrophages in diabetic nephropathy: Nrf2/HO-1 as therapeutic targets. Curr. Pharm. Des..

[62] Jin Y., Liu R., Xie J., Xiong H., He J.C., Chen N. (2013). Interleukin-10 deficiency aggravates kidney inflammation and fibrosis in the unilateral ureteral obstruction mouse model. Lab. Investig..

[63] Soranno D.E., Lu H.D., Weber H.M., Rai R., Burdick J.A. (2014). Immunotherapy with injectable hydrogels to treat obstructive nephropathy. J. Biomed. Mater. Res. Part A.

[64] Rodell C.B., Rai R., Faubel S., Burdick J.A., Soranno D.E. (2015). Local immunotherapy via delivery of interleukin-10 and transforming growth factor β antagonist for treatment of chronic kidney disease. J. Control Release.

[65] Gnudi L., Benedetti S., Woolf A.S., Long D.A. (2015). Vascular growth factors play critical roles in kidney glomeruli. Clin. Sci..

[66] Lin S., Teng J., Li J., Sun F., Yuan D., Chang J. (2016). Association of chemerin and vascular endothelial growth factor (VEGF) with diabetic nephropathy. Med. Sci. Monit..

[67] Eleftheriadis T., Antoniadi G., Pissas G., Liakopoulos V., Stefanidis I. (2013). The renal endothelium in diabetic nephropathy. Ren. Fail..

[68] Majumder S., Advani A. (2017). VEGF and the diabetic kidney: More than too much of a good thing. J. Diabetes Complicat..

[69] Schrijvers B.F., Flyvbjerg A., De Vriese A.S. (2004). The role of vascular endothelial growth factor (VEGF) in renal pathophysiology. Kidney Int..

[70] Williams T.J. (2015). Eotaxin-1 (CCL11). Front. Immunol..

[71] Wen T., Rothenberg M.E. (2016). The regulatory function of eosinophils. Microbiol. Spectr..

[72] Gauckler P., Shin J.I., Mayer G., Kronbichler A. (2018). Eosinophilia and kidney disease: More than just an incidental finding?. J. Clin. Med..

[73] Laurentius T., Raffetseder U., Fellner C., Kob R., Nourbakhsh M., Floege J., Bertsch T., Bollheimer L.C., Ostendorf T. (2019). High-fat diet-induced obesity causes an inflammatory microenvironment in the kidneys of aging Long-Evans rats. J. Inflamm..

[74] Mansouri L., Paulsson J.M., Moshfegh A., Jacobson S.H., Lundahl J. (2013). Leukocyte proliferation and immune modulator production in patients with chronic kidney disease. PLoS ONE.

[75] Akchurin O.M., Kaskel F. (2015). Update on inflammation in chronic kidney disease. Blood Purif..

[76] Klimontov V.V., Tyan N.V., Fazullina O.N., Myakina N.E., Lykov A.P., Konenkov V.I. (2016). Clinical and metabolic factors associated with chronic low-grade inflammation in type 2 diabetic patients. Diabetes Mellit..

[77] Klimontov V.V., Korbut A.I. (2019). Albuminuric and non-albuminuric patterns of chronic kidney disease in type 2 diabetes. Diabetes Metab. Syndr..

[78] Fioretto P., Caramori M.L., Mauer M. (2008). The kidney in diabetes: Dynamic pathways of injury and repair. The Camillo Golgi Lecture 2007. Diabetologia.

[79] Ekinci E.I., Jerums G., Skene A., Crammer P., Power D., Cheong K.Y., Panagiotopoulos S., McNeil K., Baker S.T., Fioretto P. (2013). Renal structure in normoalbuminuric and albuminuric patients with type 2 diabetes and impaired renal function. Diabetes Care.

[80] Robles-Osorio M.L., Sabath E. (2014). Tubular dysfunction and non-albuminuric renal disease in subjects with type 2 diabetes mellitus. Rev. Investig. Clin..

[81] Kosmas C.E., Silverio D., Tsomidou C., Salcedo M.D., Montan P.D., Guzman E. (2018). The impact of insulin resistance and chronic kidney disease on inflammation and cardiovascular disease. Clin. Med. Insights Endocrinol. Diabetes.

[82] Mihai S., Codrici E., Popescu I.D., Enciu A.M., Albulescu L., Necula L.G., Mambet C., Anton G., Tanase C. (2018). Inflammation-related mechanisms in chronic kidney disease prediction, progression, and outcome. J. Immunol. Res..

[83] Wetmore J.B., Li S., Ton T.G.N., Peng Y., Hansen M.K., Neslusan C., Riley R., Liu J., Gilbertson D.T. (2019). Association of diabetes-related kidney disease with cardiovascular and non-cardiovascular outcomes: A retrospective cohort study. BMC Endocr. Disord..

[84] Lytvyn Y., Bjornstad P., van Raalte D.H., Heerspink H.L., Cherney D.Z.I. (2020). The new biology of diabetic kidney disease-mechanisms and therapeutic implications. Endocr. Rev..

[85] Yao D., Wang S., Wang M., Lu W. (2018). Renoprotection of dapagliflozin in human renal proximal tubular cells via the inhibition of the high mobility group box 1-receptor for advanced glycation end products-nuclear factor-κB signaling pathway. Mol. Med. Rep..

[86] Heerspink H.J.L., Perco P., Mulder S., Leierer J., Hansen M.K., Heinzel A., Mayer G. (2019). Canagliflozin reduces inflammation and fibrosis biomarkers: A potential mechanism of action for beneficial effects of SGLT2 inhibitors in diabetic kidney disease. Diabetologia.

[87] Yaribeygi H., Butler A.E., Atkin S.L., Katsiki N., Sahebkar A. (2018). Sodium-glucose cotransporter 2 inhibitors and inflammation in chronic kidney disease: Possible molecular pathways. J. Cell. Physiol..

[88] Yin W., Xu S., Wang Z., Liu H., Peng L., Fang Q., Deng T., Zhang W., Lou J. (2018). Recombinant human GLP-1 (rhGLP-1) alleviating renal tubulointestitial injury in diabetic STZ-induced rats. Biochem. Biophys. Res. Commun..

[89] Chang J.T., Liang Y.J., Hsu C.Y., Chen C.Y., Chen P.J., Yang Y.F., Chen Y.L., Pei D., Chang J.B., Leu J.G. (2017). Glucagon-like peptide receptor agonists attenuate advanced glycation end products-induced inflammation in rat mesangial cells. BMC Pharmacol. Toxicol..

[90] Yaribeygi H., Maleki M., Sathyapalan T., Jamialahmadi T., Sahebkar A. (2020). Anti-inflammatory potentials of incretin-based therapies used in the management of diabetes. Life Sci..

[91] Ruiz-Ortega M., Rayego-Mateos S., Lamas S., Ortiz A., Rodrigues-Diez R.R. (2020). Targeting the progression of chronic kidney disease. Nat. Rev. Nephrol..

